# The Effect of Digestive Capacity on the Intake Rate of Toxic and Non-Toxic Prey in an Ecological Context

**DOI:** 10.1371/journal.pone.0136144

**Published:** 2015-08-19

**Authors:** Thomas Oudman, Vincent Hin, Anne Dekinga, Jan A. van Gils

**Affiliations:** Department of Marine Ecology, NIOZ Royal Netherlands Institute for Sea Research, Texel, The Netherlands; Universidad de la Republica, URUGUAY

## Abstract

Digestive capacity often limits food intake rate in animals. Many species can flexibly adjust digestive organ mass, enabling them to increase intake rate in times of increased energy requirement and/or scarcity of high-quality prey. However, some prey species are defended by secondary compounds, thereby forcing a toxin limitation on the forager’s intake rate, a constraint that potentially cannot be alleviated by enlarging digestive capacity. Hence, physiological flexibility may have a differential effect on intake of different prey types, and consequently on dietary preferences. We tested this effect in red knots (*Calidris canutus canutus*), medium-sized migratory shorebirds that feed on hard-shelled, usually mollusc, prey. Because they ingest their prey whole and crush the shell in their gizzard, the intake rate of red knots is generally constrained by digestive capacity. However, one of their main prey, the bivalve *Loripes lucinalis*, imposes a toxin constraint due to its symbiosis with sulphide-oxidizing bacteria. We manipulated gizzard sizes of red knots through prolonged exposure to hard-shelled or soft foods. We then measured maximum intake rates of toxic *Loripes* versus a non-toxic bivalve, *Dosinia isocardia*. We found that intake of *Dosinia* exponentially increased with gizzard mass, confirming earlier results with non-toxic prey, whereas intake of *Loripes* was independent of gizzard mass. Using linear programming, we show that this leads to markedly different expected diet preferences in red knots that try to maximize energy intake rate with a small versus a large gizzard. Intra- and inter-individual variation in digestive capacity is found in many animal species. Hence, the here proposed functional link with individual differences in foraging decisions may be general. We emphasize the potential relevance of individual variation in physiology when studying trophic interactions.

## Introduction

Constraints on food intake rate determine the shape of the functional response, an equation that is fundamental in population dynamical theory as it relates a forager’s intake to the density of its prey [1, 2]. The nature of these intake constraints also determines food preferences (i.e. the proportion of a prey type in the diet when not limited by availability of prey) [3, 4]. Many animals appear to be constrained by internal processing of the prey [5]. In these animals, flexibility in stomach- and/or gut size is often observed, allowing them to adjust their digestive capacity to changes in requirements and/or food availability [6–10]. However, not all food-processing pathways may be equally dependent on digestive organ size. For example, the maximum intake rate of prey with high ballast-mass may be dependent on stomach size, whereas the intake of toxic prey may be constrained by other processes that are independent of stomach size, such as the removal of toxic compounds from the body. Consequently, changing digestive organ size may not only change maximum food intake rate, but also the relative aversion for prey containing toxic compounds.

The relations between organ size, digestive capacity, prey intake rates and diet preferences have been studied step by step in experiments with red knots (*Calidris canutus*, Linnaeus) (Table 1). Red knots are medium-sized migratory shorebirds that feed on different species of mollusc prey which they ingest whole and crush in their gizzard [11–13]. Gizzard size in red knots is highly variable both between and within individuals [14, 15], and is related to the digestive quality of the diet, calculated as ash-free flesh mass over dry ballast mass [12, 16]. In captivity experiments, gizzard size can increase or decrease by 50% within one week by offering a diet of hard-shelled or soft prey items, respectively [6]. The intake rate of bivalve prey is limited by its shell-mass content as shown by van Gils *et al*. [14], who found that shell-mass processing rate relates linearly to squared gizzard mass. Since then, only two exceptions have been found to this ‘rule’. The first one is in red knots staging in the Yellow Sea, *C*. *c*. *rogersi* and *C*. *c*. *piersmai* [17], which digest the bivalve *Potamocorbula laevis* (Hinds) faster than expected from their gizzard size, probably because the force needed to crush this species is very small [18]. The second exception was found in Banc d’Arguin, Mauritania, the main wintering area of the red knot subspecies *C*. *c*. *canutus* [13, 19]. There, the most abundant mollusc prey, *Loripes lucinalis* (Lamarck), is easy to digest due to its thin shell. However, *Loripes* contains high levels of sulphur, which is produced by endosymbiotic bacteria in their gills [20]. Sulphur content of *Loripes* in Mauritania was estimated at 2–4% of dry flesh mass [21], and in such concentrations may be toxic to any animal species [22].

**Table 1:** Experimental studies on gizzard size and diet in red knots.

Study result	Reference
Gizzard size is related to diet	Piersma *et al*. [12]
Gizzard size responds to changes in diet	Dekinga *et al*. [6]
Shell-mass processing rate is a function of gizzard size	van Gils *et al*. [14]
Shell-mass processing rate explains diet preferences	van Gils *et al*. [16]
Shell-mass processing rate is higher on easy-to-crush prey	Yang *et al*. [18]
Maximum intake on toxic prey not set by shell-mass processing rate	Oudman *et al*. [23]

Oudman *et al*. [23] showed experimentally that red knots foraging *ad libitum* on *Loripes* are limited by the presumably toxic concentration of sulphur rather than by shell-mass processing rate. This toxic effect also explained the observed prey preferences, both in the laboratory [23] and in the field [24]. Whereas red knots *C*. *c*. *islandica* in the Wadden Sea are solely limited by shell-mass processing rate and always preferred the prey with the highest digestive quality [16], *C*. *c*. *canutus* in Mauritania preferred a mixed diet of toxic but easy-to-digest *Loripes* and *Dosinia isocardia* (Dunker), the latter which is harder to digest but not toxic [23, 24]. The preferred proportion of *Loripes* in the diet appeared to depend on the strength of the toxin constraint relative to the digestive constraint. Hence, if gizzard size changes digestive capacity but not detoxification rate, the preference for *Loripes* is expected to be higher in birds with a small gizzard than in birds with a large gizzard.

In this study we tested (1) whether the maximum intake rate of sulphur-containing *Loripes* is indeed independent of gizzard size, and (2) whether maximum intake rate of *Dosinia* matches the earlier observed linear relation with squared gizzard size. This was done by manipulating gizzard sizes of 6 captive red knots in Mauritania through prolonged diets of either soft or hard-shelled prey, and afterwards measuring intake rates on both prey species in separate trials. Subsequently, the procedure was repeated with the soft- and hard-shelled diets reversed. In the discussion section we extend a linear programming model [3, 4, 25] that is described in Oudman *et al*. [23], to quantify the expected diet preferences as a function of gizzard size.

## Methods

### Birds and gizzard manipulation

The experiment was performed at the Iwik research station located on the peninsula of Iwik in the Banc d’Arguin, Mauritania. Six adult red knots were caught using mist nets on the night of 20 January 2012 and ringed with unique combinations of colour-rings for identification. Birds were held in an indoor cage (1.5 x 1 x 0.5 m) in a room with windows, and temperatures varying between 18 and 22°C. Food availability outside experimental trials was adjusted to maintain a low but not unnatural body mass (between 100 and 110 g) [26]. Together with food deprivation for at least 2 h before each trial, this ensured that all birds were motivated to feed during the experimental trials. Gizzard masses were non-invasively measured regularly using ultrasonography [6, 27] (for more details see S1 File).

Birds were randomly divided into two groups of three birds with each group receiving a different gizzard-manipulating food regime outside the experimental trials. Initial differences in gizzard mass between groups were not significant (F_1,4_ = 3.9, p = 0.12). Group 1 received hard-shelled prey to maintain a large gizzard. Prey for this group were collected on the sandy beach near the research station and consisted mainly of *Dosinia isocardia* but also small *Senilia senilis* and *Bittium reticulatum*. Additionally, flesh of large *Senilia senilis* was provided because not enough hard-shelled prey could be collected to satisfy the energy demands of the birds. Group 2 was provided only with flesh of *Senilia senilis*, which is a food type that decreases gizzard mass [6]. Outside the experimental trials birds had constant access to freshwater. Fourteen days after the birds had been caught a first series of experimental trials was performed spread over a period of ten days. After this period, the food regimes outside the trials were reversed between the groups, now with group 1 being provided soft food and group 2 a mixture of hard-shelled prey and soft food. Seven days after the reversal, a second series of experimental trials was performed over a period of eight days.

### Experimental design

The experiment comprised a total of 60 trials. The first series of trials (thus before the gizzard-manipulation reversal) consisted of 39 trials, measuring intake rate of isolated birds either on *Dosinia isocardia* (3 or 4 trials per bird, 19 in total) or *Loripes lucinalis* (3 or 4 trials per bird, 20 in total). In the second series (thus after the gizzard-manipulation reversal), two *Dosinia* trials and two *Loripes* trials were performed with each bird (24 trials in total). During the second series of trials, one bird in group 2 started showing general signs of illness such as improper preening, ruffled feathers, reduced feeding and docile behaviour. The trials of this bird after the onset of illness (3 trials: 2x *Loripes* diet, 1x *Dosinia* diet) were removed from the intake-rate analysis as well as from the gizzard-mass analysis. *Dosinia* and *Loripes* were gathered daily in a sieve (mesh size 2 mm) from a sandy beach and a seagrass bed, respectively. Bivalves were offered alive one day after gathering. During each trial, food (either *Dosinia* or *Loripes*) and seawater was provided *ad libitum* for two hours, during which total intake was measured.

We estimated the number and size distribution of the eaten prey items by counting and measuring shell lengths of a sub-sample of each species to the nearest 1 mm at the start and at the end of each trial. Each sub-sample consisted of 100 prey items, or all prey items if less than 100 prey were left after the trial. Size distribution was estimated in length classes of 1 mm. To determine length-specific dry mass of shell (DM_shell_) and ash-free dry mass of flesh (AFDM_flesh_), 100 individuals of each prey species were stored in 4% borax-buffered formalin before analysis at the NIOZ Royal Netherlands Institute for Sea Research. Length of each individual was measured to the nearest 0.1 mm, after which flesh and shell were dried separately at 60°C for 3 days, weighed, incinerated at 560°C for 5 hours (only the flesh) and weighed again. The estimated number of ingested prey items in each size-class was multiplied by its estimated DM_shell_ to arrive at an estimation of total ingested DM_shell_. These estimates were compared with measured dry-mass of the faeces produced from the start until 4 hrs after the end of each trial. Pooling all before-trial shell measurements per species and setting negative estimations of the eaten number of prey in a size class to zero (which occurred only in the rare length classes) improved the correlation with dry faeces mass from 0.81 (Pearson’s coefficient, t = 11.7, df = 69, p < 0.001) to 0.84 (t = 13.0, p < 0.001).

### Statistical analysis

Statistics were performed in R version 3.1.0 [28]. The effects of group (group 1 or group 2) and diet (soft prey or hard-shelled prey) on gizzard mass during the experimental trials were tested by AIC_c_ comparison (function “aictab” in package “AICcmodavg”) of linear mixed-effects models (function “lme” in package “nlme”), estimating parameter values by maximizing log-likelihood [29]. Bird-ID was included in each model as a random effect. Trends in the rate of change in gizzard mass from catch until the end of the experiments were analysed by local regression (function “loess”, span = 0.5) on 13–16 measurements for each bird spread over the whole period. These regressions were used to estimate gizzard mass during each particular experimental trial.

The effects of gizzard size (large or small gizzard) and prey species (either *Dosinia* or *Loripes*) on intake rate in the experiment were tested by AIC_c_ comparison of linear mixed-effect models, including Bird-ID as a random effect. A variance structure was incorporated to correct for different variances in *Dosinia* and *Loripes* intake rates. *Dosinia* had a larger size range (3–15 mm) than *Loripes* (4–12 mm), and as larger bivalves contained exponentially more shell and flesh, estimations of DM_shell_ eaten from larger size classes gave exponentially larger variances. For *Loripes* as well as for *Dosinia*, the relation between DM_shell_ and shell length was estimated with a local regression function (function “loess”, span = 0.6), as non-linear regression did not give a satisfying fit (for details see S2 File) [30].

### Ethics statement

The experiment was performed under full permission by the authorities of the Parc National du Banc d’Arguin (PNBA). No animal experimentation ethics guidelines exist in Mauritania. However, the experiment was carried out in strict accordance with Dutch animal experimentation guidelines. The NIOZ Royal Netherlands Institute for Sea Research has been licensed by the Dutch Ministry of Health to perform animal experiments under license number 80200. This license involves capture and handling of animals, and performing experiments, which nonetheless should be individually approved by the Animal Experimentation Committee (DEC) of the Royal Netherlands Academy of Arts and Sciences (KNAW). The DEC does not provide permits for experiments in foreign countries, but provided approval for equivalent experiments in the Netherlands by the same persons under permit number NIOZ 10.05, involving the capture of red knots, performing non-invasive experiments consisting of prolonged diets of natural food types (i.e. foods that regularly occur in the diet of wild red knots) and repeated gizzard size measurements, and includes permission to release healthy animals in the wild after the experiment.

All possible efforts were made to minimize physical and mental impact on the experimental animals. Each bird was weighed and visually inspected for general condition daily, and removed from the experiment when not healthy (one bird). The reasons for the experiment to take place in Mauritania were purely scientific and by no means to avoid ethics guidelines. All experimental animals were released in the wild in healthy condition after the experiment.

## Results

**Fig 1:**
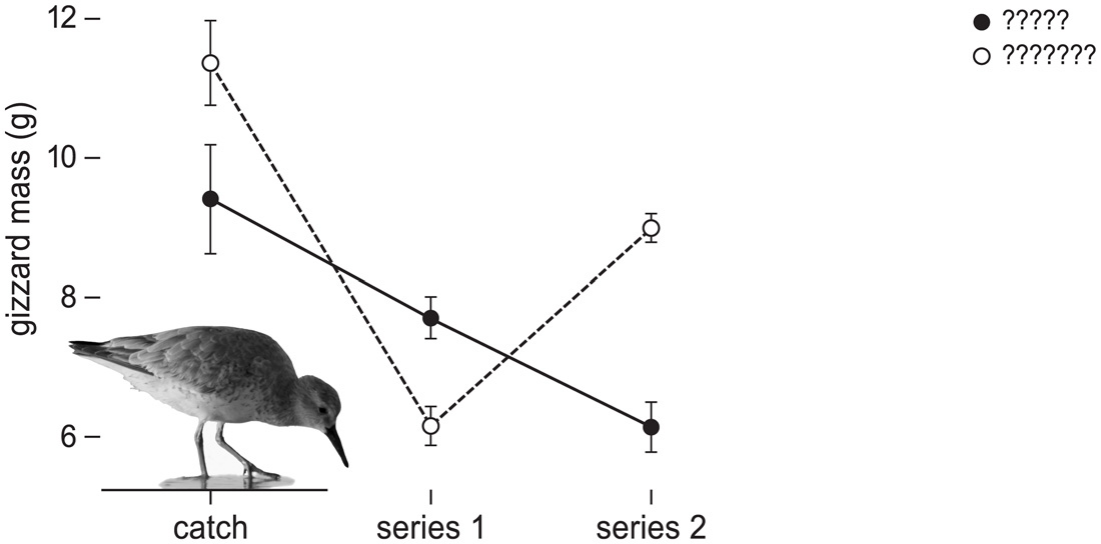
Mean gizzard mass of birds directly after catch, during the first and second series of trials. Directly after catch, the 6 red knots were randomly divided into two groups, group 1 (solid dots and line) and group 2 (open dots and dashed line). Both groups received different diets outside the experimental trials (soft or hard-shelled prey) to manipulate gizzard size. Initial differences in gizzard mass between groups were not significant (F_1,4_ = 3.9,p = 0.12). After catch, all birds decreased gizzard mass, but group 1 had larger gizzards than group 2 during the first series of trials, and smaller gizzards during the second series (Table 2, models 1.1 to 1.5), showing that the manipulation of gizzard size was successful. Each group consisted of three birds. However, data collected on one bird from group 2 after it became sick during series 2 was omitted from the graphs and the analysis. Error bars show standard error.

The diet treatments successfully resulted in gizzard mass changes in the experimental red knots (Fig 1, model comparison in Table 2, see S1 Table for model estimates). Although all birds initially reduced gizzard mass, a diet of hard-shelled prey resulted in larger gizzards (estimate±SE: 8.3±0.3 g) than soft prey (6.1±0.3 g). Group (group 1 or group 2) had no significant effect on gizzard mass. However, the hard-shelled diet led to a larger rate of gizzard mass increase in group 2 than in group 1 (significant interaction between diet and group, see Table 2 and model estimates in S1 Table), presumably because the birds were less eager to increase gizzard mass soon after the catch (see also Fig 1). The diet-induced rates of change in gizzard mass where comparable to those found earlier (for details see S3 File) [6].

**Table 2:** Second-order Akaike’s information criterion (AIC_c_) comparison of statistical models.

Model	Fixed effects^a^	K^b^	ΔAIC_c_	AIC_c_ weight	Cumulative weight	LL^c^
Response variable: Gizzard mass						
1.1	Diet × group	6	-	0.69	0.69	-81.2
1.2	Diet	4	2.90	0.16	0.85	-85.2
1.3	Diet + group	5	3.00	0.15	1	-84.1
1.4	1	3	44.23	0	1	-107.1
1.5	Group	4	44.89	0	1	-106.2
Response variable: DM_shell_ ^d^ intake rate of *either Loripes or Dosinia*						
2.1	Gizzard × species	7	-	0.96	0.96	-40.4
2.2	Species	5	7.45	0.02	0.98	-46.7
2.3	Gizzard + species	6	7.76	0.02	1	-45.6
2.4	1	4	35.52	0	1	-61.9
2.5	Gizzard	5	37.09	0	1	-61.5
Response variable: log transformed DM_shell_ intake rate						
3.1	Log(gizzard)	4	-	0.85	0.85	-17.07
3.2	Log(gizzard) + species	6	4.34	0.10	0.95	-16.86
3.3	Log(gizzard) × species	8	5.65	0.05	1	-14.96
3.4	species	5	20.53	0	1	-26.17
3.5	1	3	22.11	0	1	-29.26

Model selection based on AIC_c_, with a penalty of 2 per added parameter [29]. Models are ordered by adequacy, starting with the minimum adequate model. Model 1.2 is competitive with model 1.1. Model 2.1 and 3.1 do not have competitors. All models are linear mixed models with a Gaussian error structure, and contain bird ID as a random effect. Models 2.1 to 2.5 contain a variance structure based on prey species.

^a^ In model 1.1 to 1.5, factor “diet” refers to the diet outside the experimental trials, being either soft or hard-shelled. Factor “group” refers to the order of these diet treatments (group 1 or group 2). In models 2.1 to 2.5, factor “gizzard” refers to gizzard size during the trial, which was either small or large; “species” refers to the prey species being offered, which was either *Dosinia* or *Loripes*. In models 3.1 to 3.5 log(gizzard) is a continuous variable that refers to the logarithm of estimated gizzard mass during the trial; species refers to prey species, which was either *Dosinia isocardia*, *Cerastoderma edule* or *Macoma balthica*. The symbol × means that the main terms as well as their interaction are fixed effects in the model. Models 1.4, 2.4 and 3.5 contain only an intercept, no fixed effects.

^b^ The number of parameters in the model.

^c^ Log likelihood.

^d^ Dry ballast mass.

**Fig 2:**
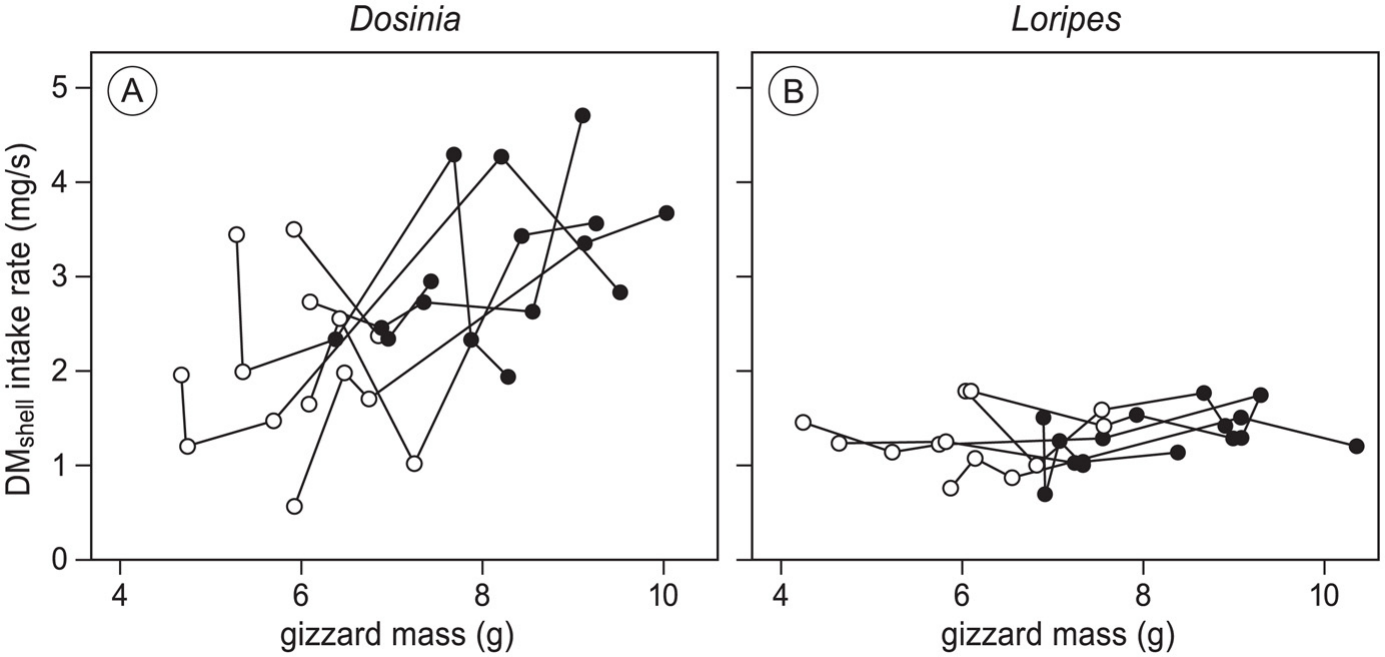
Dry shell mass (DM_shell_) intake rate on a *Dosinia* diet (A) and on a *Loripes* diet (B). Lines connect all trials of the same bird when it was in the small gizzard group (open dots) and in the large gizzard group (solid dots). Intake of *Dosinia* was higher for birds with large gizzards, whereas intake of *Loripes* was not affected by gizzard size (model 2.1 in S1 Table). *Loripes* intake rate was generally lower than *Dosinia* intake rate. These results confirm that intake of *Dosinia* is limited by a digestive constraint, whereas intake of *Loripes* is limited more stringently, presumably by its toxic load, and independent of gizzard mass.

Gizzard mass manipulations had an effect on intake rate (expressed as DM_shell_), dependent on prey species (model 2.1 in Table 2, see S1 Table for model estimates). As expected, DM_shell_ intake of toxic *Loripes* did not change with an increase in gizzard mass (estimated change from 1.25 to 1.31 mg/s, t = 0.65, p = 0.52), whereas intake of non-toxic *Dosinia* did increase with gizzard mass (estimated change from 2.00 to 3.12 mg/s, t = 3.73, p<0.001). DM_shell_ intake on a *Loripes* diet was lower than on a *Dosinia* diet for small gizzard birds (estimated difference -0.75 mg/s, t = -3.21, p = 0.002) as well as for large gizzard birds (estimated difference -1.81 mg/s, t = -8.37, p<0.001). These results are depicted in Fig 2, where gizzard masses are shown on a continuous scale. The results indicate that the shell-mass processing constraint was alleviated with an increase in gizzard mass, as predicted, and that the toxin constraint was independent of gizzard mass. To test if morphological characters of individual birds other than gizzard size influenced intake rate, the explanatory variables body mass, bill length, tarsus length and wing length of the individual birds were separately added to model 2.1. None of these variables improved the statistical fit of the model (results not shown).

## Discussion

### Maximum intake rate as a function of gizzard mass

**Fig 3:**
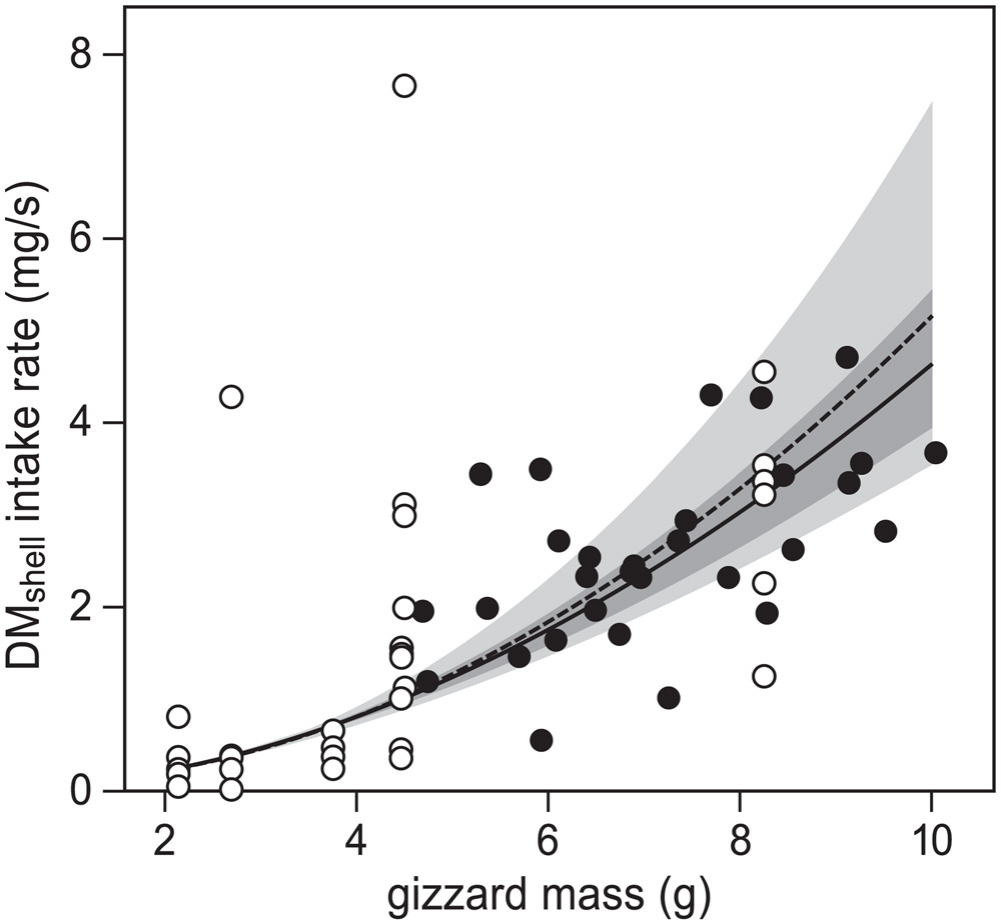
Regression of DM_shell_ intake on non-toxic prey against gizzard mass. Data from this study on *Dosinia* was combined with data from van Gils *et al*. [14] on other non-toxic prey species. Adding the current data to the regression derived by van Gils *et al*. [14] slightly changes the regression line (though not significantly; from dashed to solid line), but greatly reduces standard error (from light to dark grey area). Parameter estimates are shown in S1 Table (model 3.1). Regressions are linear regressions on log-transformed data. Note that van Gils *et al*. [14] averaged gizzard mass measurements per bird, whereas we estimated gizzard mass in each trial by interpolating measurements.

To confirm that the relation between gizzard mass and dry shell-mass (DM_shell_) intake rate on *Dosinia* agreed with the earlier observed relations by van Gils *et al*. [14], we compared the two outcomes. Van Gils *et al*. measured maximum DM_shell_ intake rates in 6 captive red knots (*C*. *c*. *islandica*) in the Dutch Wadden Sea on two non-toxic bivalve species, *Cerastoderma edule* (Linnaeus) and *Macoma balthica* (Linnaeus). Similar to the present study, they manipulated gizzard masses by placing birds randomly in one of two groups, one with a soft prey diet and the other with a hard-shelled diet. They estimated gizzard mass in each bird as the mean of a series of gizzard measurements in the course of the experimental trials. By comparing linear models, they concluded that DM_shell_ intake was independent of bird individual, prey species and prey size. They found a linear relationship with gizzard mass on log-transformed data (R^2^ = 0.48, p < 0.001, Fig 3).

The effect of gizzard mass on prey intake rate, and a potential difference between the two studies on this relation was tested by combining both datasets, and comparing AIC_c_ values of linear mixed-effect models on log-transformed data (models 3 in Table 2), containing bird-ID as random effect. As expected, the model that best explained DM_shell_ intake rate did not include prey species (*Dosinia*, *Cerastoderma* and *Macoma*; model 3.1 in Table 2, see S1 Table for model estimates), but did contain gizzard mass in the following way:

$$c=10^{-1.244}G^{1.9}\;,$$ (1)

where *c* is DM_shell_ intake rate (mg/s) and *G* is gizzard mass (g). This estimated relation does not differ from *c* = 10^−1.293^ *G* ^2.0^ as found by van Gils *et al*. [14], as standard errors completely overlap (Fig 3).

### Within- and between-year variation in the toxin constraint

Maximum intake rate of *Loripes* in this study did not differ between large- and small-gizzard birds (Fig 2). Because sulphur, presumably the toxic compound in *Loripes*, resides in the flesh and not the shell, we will from here on refer to the toxin constraint in terms of ash-free dry flesh mass (AFDM_flesh_) instead of DM_shell_. The best estimate of AFDM_flesh_ intake rate is given by an intercept mixed-effect model on the *Loripes* data, with bird-ID as a random effect, giving an estimate of 0.21 mg/s, with a within-individual variance of 0.002 and a between-individual variance of 0.0005 [31]. One year earlier, the intake constraint on *Loripes* was estimated at 0.12 mg/s [23], with a within-individual variance of 0.0003 and a between-individual variance of 0.001 (Oudman unpublished data). The large difference between the two years in the intake constraint, despite small within- and between-individual variances within each year, is remarkable. This difference may be explained by yearly variation in the toxic load of *Loripes*, and/or by a difference in the capability or costs paid by red knots to deal with the toxic load of *Loripes*. The high consistency in *Loripes* intake between birds within years favours the first explanation. Differences in toxic load may relate to the mixotrophic life style of *Loripes* [32] and potentially has effects on the spatial distribution and population dynamics of *Loripes*, by influencing predation risk [33, 34].

### (In)flexibility of the toxin constraint

Most of the mollusc biomass available to red knots in Banc d’Arguin consists of *Loripes* [33, 35–37], but its observed proportion in the diet is low [24, 33, 37, 38]. Hence, releasing the toxin constraint would likely enable red knots to increase energy intake rate or decrease required foraging time in the field. The physiological processes that make *Loripes* toxic to red knots have not been studied, but may involve sulphide formation in the intestines during digestion. Most vertebrates can detoxify sulphide to a limited extent by oxidation to sulphate in the mitochondria of liver cells and red blood cells, and excretion by the kidney [39, 40]. Energy investment in these detoxification pathways may enable red knots to increase their sulphur tolerance, but the consistent low fraction of *Loripes* in the diet and the low individual variation in the toxin constraint (see also [23]) suggests that sulphur tolerance either cannot be adjusted or is very costly to increase.

### Diet preferences as a function of gizzard size

**Fig 4:**
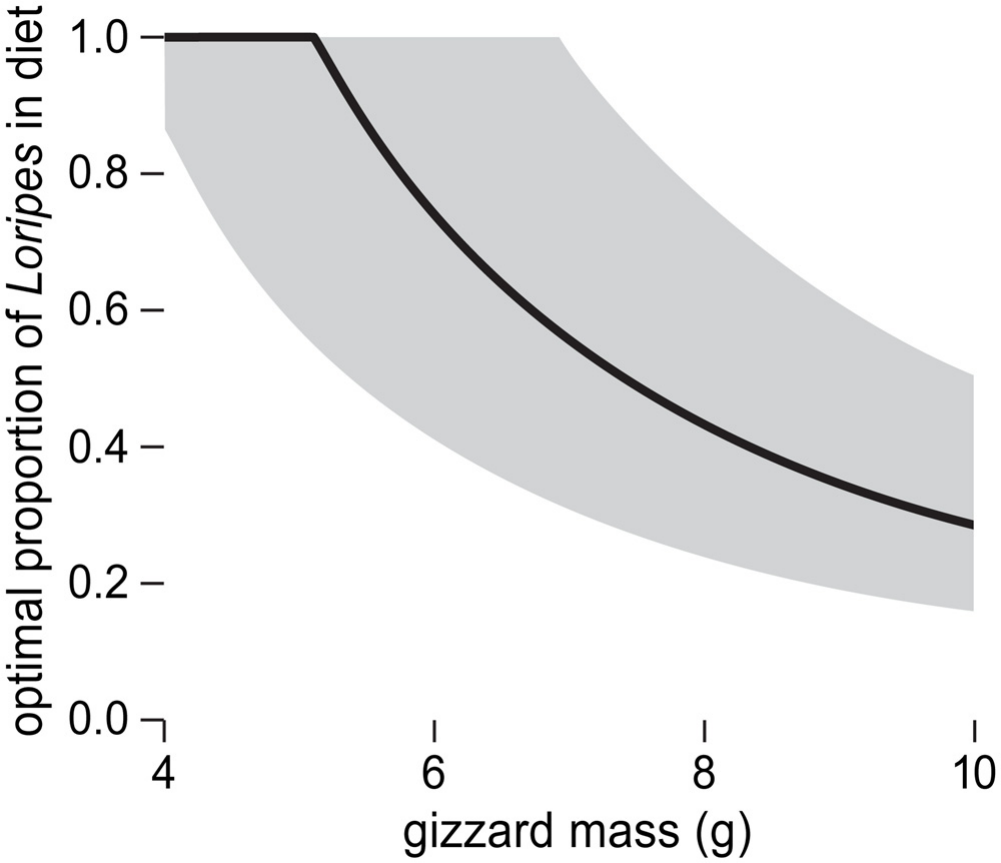
The predicted optimal proportion of *Loripes* in terms of dry shell mass in the diet of an energy intake maximizing red knot that has *ad libitum* access to both *Loripes* and *Dosinia*. Red knots with small gizzards are expected to feed exclusively on *Loripes*, whereas red knots with large gizzards are expected to have a large share of *Dosinia* in the diet. Grey area shows 95% prediction interval. See S4 File for more details.

Gizzard masses of red knots caught in Banc d’Arguin are variable between individuals (mean = 9.89 g, SD = 1.30 g; [15]), ranging from 4 to 15 g (A. Dekinga unpublished data). These differences in gizzard mass may accompany differences in diet preferences, as gizzard mass influences potential intake on *Dosinia* but not on *Loripes*. Linear programming models can be used to quantify optimal diet preferences as a function of the constraints on intake rate under the assumption of energy maximization [3, 4, 25]. Oudman *et al*. [23] use a linear programming model to calculate expected diet preferences for energy intake maximizing red knots foraging on *ad libitum Loripes* and *Dosinia*. This model calculates which combinations of intake rates on *Dosinia* and *Loripes* are possible given both the shell-mass processing constraint and the toxin constraint on *Loripes*, and subsequently determines which of these combinations provides the highest energy intake rate. Based on measured values of the shell-mass processing constraint and the toxin constraint on *Loripes* but without taking gizzard mass into account, they deduce that the optimal proportion of *Loripes* in the diet is 39% in terms of dry shell mass, when both prey occur in *ad libitum* abundances. van Gils *et al*. [24] show how this optimal proportion varies with densities of both prey. Replacing a constant shell-mass processing constraint by the here derived gizzard-mass dependent shell-mass processing constraint (eq. 1) and parameterizing the model with the here obtained values (for a detailed model description, see S4 File) shows that this proportion changes considerably with gizzard mass (Fig 4). The model predicts that energy maximizing birds with a gizzard mass below 5.2 g prefer an exclusive *Loripes* diet. Red knots with greater gizzard masses are expected to have a lower proportion of *Loripes* in the diet, which is less than 40% of total DM_shell_ intake rate in birds with a 10 g gizzard. Hence, model predictions show that given the observed variation in gizzard sizes of red knots in the wild, considerable inter- and intra-individual variation in diet preferences can be expected. This result may translate to many other species, because flexibility in digestive organ mass is a general phenomenon [41] being observed in mammals [42], reptiles [9], fish [8] and birds [10]. Toxin constraints are observed widely too, especially in herbivores, e.g. [43], but are not a prerequisite to explain a functional link between individual variation in physiology and diet preferences. For example, external handling constraints may also, in combination with digestive capacity, cause a mixed diet that depends on the strength of the digestive constraint [25].

To experimentally test the here predicted link between digestive capacity and diet preferences comes with complications. If the animals adjust their preferences to gizzard mass in an experiment with gizzard manipulations, it is clear that they base their choice on physiological state. However, if the animals do not adjust their preferences, the here predicted link may still be correct, but the causality reversed; in that case, gizzard mass may be adjusted to individual differences in diet [44]. Hence, the model cannot be proven incorrect in the experimental setting presented in this paper, but should be accompanied by field observations.

## Supporting Information

S1 Dataset (XLSX)

S1 File Gizzard mass measurements. (PDF)

S2 File Estimating dry shell mass from shell length. (PDF)

S3 File Rates of change in gizzard mass. (PDF)

S4 File Predicting diet preferences from gizzard mass: a linear programming model. (PDF)

S1 Table Parameter estimates in fixed part of minimum adequate statistical models. (PDF)
