# Supplementary material for: The Effect of Digestive Capacity on the Intake Rate of Toxic and Non-Toxic Prey in an Ecological Context
Source: PLoS One. 2015 Aug 19;10(8):e0136144. doi: 10.1371/journal.pone.0136144 (PMC4543589; doi:10.1371/journal.pone.0136144)
Supplement: S1 File — (PDF) [file pone.0136144.s002.pdf]

S1 File. Gizzard mass measurements

Gizzard size of each bird was measured within one day after catch, and every third day during both series of trials. They were measured non-invasively by AD and TO with an ultrasound apparatus (model Aquilla, Pie Medical Benelux, Maastricht, The Netherlands), according to the procedure described in Dekinga et al. [1]. The observer did not know to which experimental group each bird belonged. Height (H) and width (W) were always measured twice and both averaged. Average H and W were transformed to gizzard mass (G) by the formula $G = -1.09 + 3.78HW$, derived in a calibration study on 29 dead red knot bodies with variable gizzard masses (A.D., unpublished data). Gizzard mass estimations did not differ between AD and TO when repeated by both observers (n = 35). The slope of the major axis regression (function “sma” in R package “smatr”) did not differ from 1 (95%CI [0.96,1.66], r = 0.28, p = 0.1) and the elevation did not differ from zero (95%CI [-4.97,0.48], t = -1.6, p = 0.1). Gizzard mass on each day was modelled for each bird with a polynomial model, fitted to all measurements (function “loess” in the basic package in R, span = 0.5).

References

1. Dekinga A, Dietz MW, Koolhaas A, Piersma T. Time course and reversibility of changes in the gizzards of red knots alternately eating hard and soft food. Journal of Experimental Biology. 2001;204(12):2167-73.
