# Supplementary material for: The Effect of Digestive Capacity on the Intake Rate of Toxic and Non-Toxic Prey in an Ecological Context
Source: PLoS One. 2015 Aug 19;10(8):e0136144. doi: 10.1371/journal.pone.0136144 (PMC4543589; doi:10.1371/journal.pone.0136144)
Supplement: S2 File — (PDF) [file pone.0136144.s003.pdf]

S2 File. Estimating dry shell mass from shell length

Allometric relations are classically estimated as power functions of the form $Y = aX^b$ [1]. When this method is applied to the relation between shell length and shell dry mass (DM_{shell}) in *Loripes* and *Dosinia*, DM_{shell} of individuals between 8 and 10 mm are underestimated (see Fig. S2.1). The exponent of the allometric equation appears to rise after 8 mm of length. This appears to be a general tendency in bivalves [2-4]. Therefore, we expect the inflected curve to be a consequence of the ontogeny of bivalves. Fitting a loess function instead of a power function accounts for the changing exponent [4].

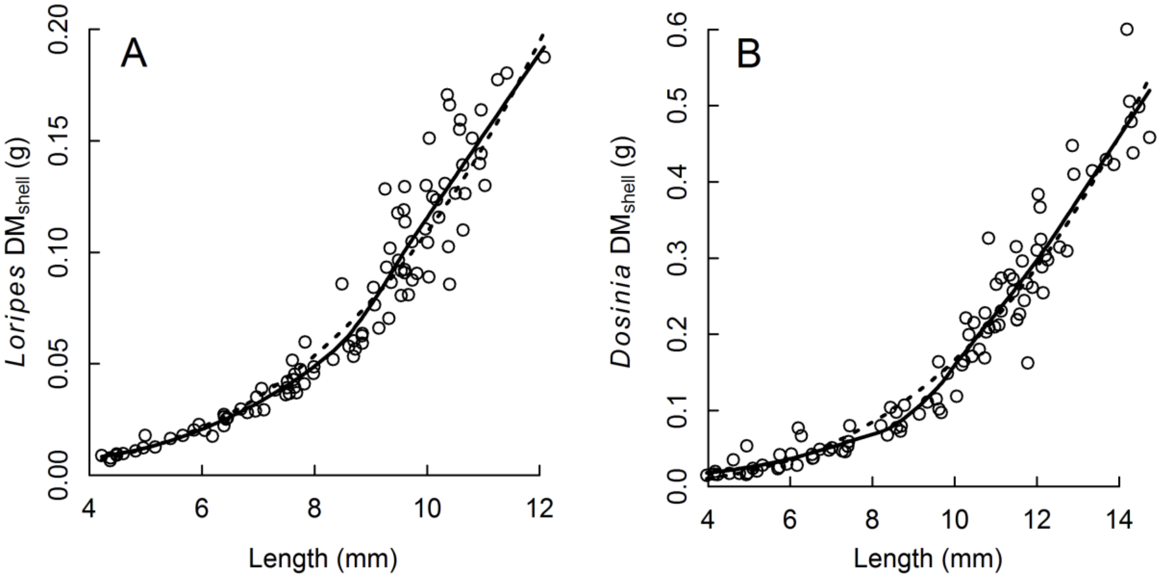

Figure S2.1. Dry shell mass (DM_{shell}) as a function of length for *Loripes* (A) and *Dosinia* (B). Fitting a power curve (dashed line) gives an overestimation of DM_{shell} in medium sized (8-10 mm) individuals, in both prey species. Fitting a loess curve (span = 0.6) solves this issue (solid line). Note the different scalings of the axes.

References

1. Huxley J. Problems of relative growth. London: Methuen; 1932.
2. Katsanevakis S, Thessalou-Legaki M, Karlou-Riga C, Lefkaditou E, Dimitriou E, Verriopoulos G. Information-theory approach to allometric growth of marine organisms. Mar Biol. 2007;151(3):949-59. doi: 10.1007/s00227-006-0529-4. PubMed PMID: WOS:000246098900012.
3. Hendriks IE, Basso L, Deudero S, Cabanellas-Reboredo M, Alvarez E. Relative growth rates of the noble pen shell *Pinna nobilis* throughout ontogeny around the Balearic islands (western Mediterranean, Spain). Journal of Shellfish Research. 2012;31(3):749-56. doi: 10.2983/035.031.0319.
4. Bijleveld AI, Twietmeyer S, Piechocki J, van Gils JA, Piersma T. Natural selection by pulsed predation: survival of the thickest. Ecology. 2015;96(7):1943–56.
