# Supplementary material for: The Effect of Digestive Capacity on the Intake Rate of Toxic and Non-Toxic Prey in an Ecological Context
Source: PLoS One. 2015 Aug 19;10(8):e0136144. doi: 10.1371/journal.pone.0136144 (PMC4543589; doi:10.1371/journal.pone.0136144)
Supplement: S3 File — (PDF) [file pone.0136144.s004.pdf]

S3 File. Rates of change in gizzard mass

Initially, all birds decreased gizzard mass after catch (mean ± between-individual SE: -0.40±0.09 g/day). After reaching a minimum around day 10 after catch, group 1 birds slightly increased gizzard mass again (0.23±0.02 g/day), whereas group 2 on average remained stable (0.04±0.08 g/day). After the diet switch at day 24, group 1 decreased gizzard mass (-0.24±0.02 g/day) whereas gizzard masses of group 2 increased (0.30±0.06 g/day). The observed rate of diet-related gizzard mass increase was identical to the rate observed by Dekinga *et al*. [1] who found a diet-induced rate of increase of 0.30±0.05 g/day. The diet-induced rate of decrease was slightly weaker in this study than in Dekinga *et al*. (-0.38 g/day, SE not given), which however fits well with the here observed initial decrease rate after catch.

References

1. Dekinga A, Dietz MW, Koolhaas A, Piersma T. Time course and reversibility of changes in the gizzards of red knots alternately eating hard and soft food. Journal of Experimental Biology. 2001;204(12):2167-73.
