# Supplementary material for: The Effect of Digestive Capacity on the Intake Rate of Toxic and Non-Toxic Prey in an Ecological Context
Source: PLoS One. 2015 Aug 19;10(8):e0136144. doi: 10.1371/journal.pone.0136144 (PMC4543589; doi:10.1371/journal.pone.0136144)
Supplement: S4 File — (PDF) [file pone.0136144.s005.pdf]

S4 File. Predicting diet preferences from gizzard mass: a linear programming model

A situation is assumed in which both *Loripes* and *Dosinia* are offered *ad libitum* to red knots that are maximizing energy intake rate. The idea of the linear programming model is to first derive all possible combinations of dry shell mass intake rates on *Dosinia* and *Loripes* (r_d and r_l, measured in mg/s; see Table S4.1 for a list of all used symbols) while respecting both the ballast-mass processing constraint and the toxin constraint (Fig. S4.1 A). Then it is determined which of all possible combinations of r_d and r_l provides the highest energy intake rate, denoted as $R^* = (r_d^*, r_l^*)$. Oudman *et al.* [1] deduced that as long as *Loripes* is limited by a toxin constraint, R^* is found by drawing both constraints in a plane spanned by r_d and r_l. R^* is the point where both constraint lines intersect. It is calculated as:

$$(r_d^*, r_l^*) = (c - q, q), \qquad (S1)$$

where *c* is the digestive constraint (maximum dry shell-mass (DM_{shell}) intake in mg/s); *q* is the toxin constraint (maximum DM_{shell} intake *Loripes* in mg/s). The units differ from Oudman *et al.* [1], where intake rates were measured in individuals per second. Instead, we measured intake rate in mg DM_{shell} per second to facilitate the implementation of the current experimental results, where prey of variable sizes were used. We can do so because no relation between prey length and the ratio of ash-free dry flesh-mass over dry shell mass ($AFDM_{flesh}$:DM_{shell}) was found, neither in *Dosinia* ($R^2 = 0.006$, $p = 0.22$) nor in *Loripes* ($R^2 = 0.005$, $p = 0.22$). Energy content of *Dosinia* and *Loripes* (e_d and e_l), measured as $AFDM_{flesh}$ per unit of DM_{shell}, was estimated for both *Dosinia* and *Loripes* by averaging all measured individuals without accounting for size, resulting in 0.057±0.001 (mean±SE) and 0.163±0.005 mg $AFDM_{flesh}$ per mg DM_{shell}, respectively.

The optimization procedure can be performed graphically by drawing both constraints as lines in a plane spanned by r_d and r_l. In each point in this plane, total intake rate of ash-free flesh mass can be calculated by for each prey species multiplying DM_{shell} intake rate with energy content, and adding them up:

$$Y = r_d e_d + r_l e_l. \qquad (S2)$$

The optimal combination of r_d and r_l (R^*) is found by maximizing Y, given that neither constraint line is crossed. Fig. S4.1 B shows that changing gizzard mass from 6 g to 9 g leads to an increase in the digestive constraint, but not the toxin constraint. Fig. S4.1 B shows the constraint lines both for a 6 and a 9 g gizzard in the plane spanned by r_d and r_l, showing a shift in r_d^* but not in r_l^*. Hence, the absolute amount of *Loripes* in the diet remains constant, but the proportion of *Loripes* in the diet decreases when gizzard mass increases (Fig S4.1 C).

The relation between gizzard mass and R^* can be formalized by inserting equation 1 from the main text, denoting *c* as a function of gizzard mass *G* (g), into equation S1. Contrastingly, *q* is constant and estimated as 1.29 mg DM_{shell} per second (linear mixed-effect intercept model on *Loripes* data, containing bird-ID as random effect). Hence, R^* is dependent on gizzard mass in the following way:

$$(r_d^*, r_l^*) = (10^{-1.244} G^{1.9} - 1.29, 1.29). \qquad (S3)$$

When gizzard mass drops below 5.2 g, then *Loripes* intake rate is no longer limited by the toxin constraint, but becomes limited by the shell-mass processing constraint. In that case r_d^* becomes zero (see [1] for more details). The expected diet preferences, which we define as

the optimal proportion of *Loripes* in the diet, is calculated by dividing r_l^* by total DM$_{shell}$ intake:

$$\frac{r_l^*}{r_d^* + r_l^*} = \frac{22.6}{G^{1.9}} \quad if \quad G > 5.2g \qquad (S4a)$$

$$\frac{r_l^*}{r_d^* + r_l^*} = 1 \qquad otherwise \qquad (S4b)$$

This relation is shown in Fig. S4.1 C. In conclusion, red knots with a gizzard below 5.2g are expected to always prefer *Loripes* over *Dosinia*, and birds with larger gizzard sizes to include a proportion of *Dosinia* in their diet that increases with gizzard size. The uncertainty in the predicted preferred diet that results from the variances in the constraint measurements was relatively large (grey area in Fig. S4.1 C), as they are multiplied in the estimation. The prediction interval was calculated by drawing 100.000 values for each of a sequence of gizzard masses from simulated constraint values, which were assumed to follow the normal distribution.

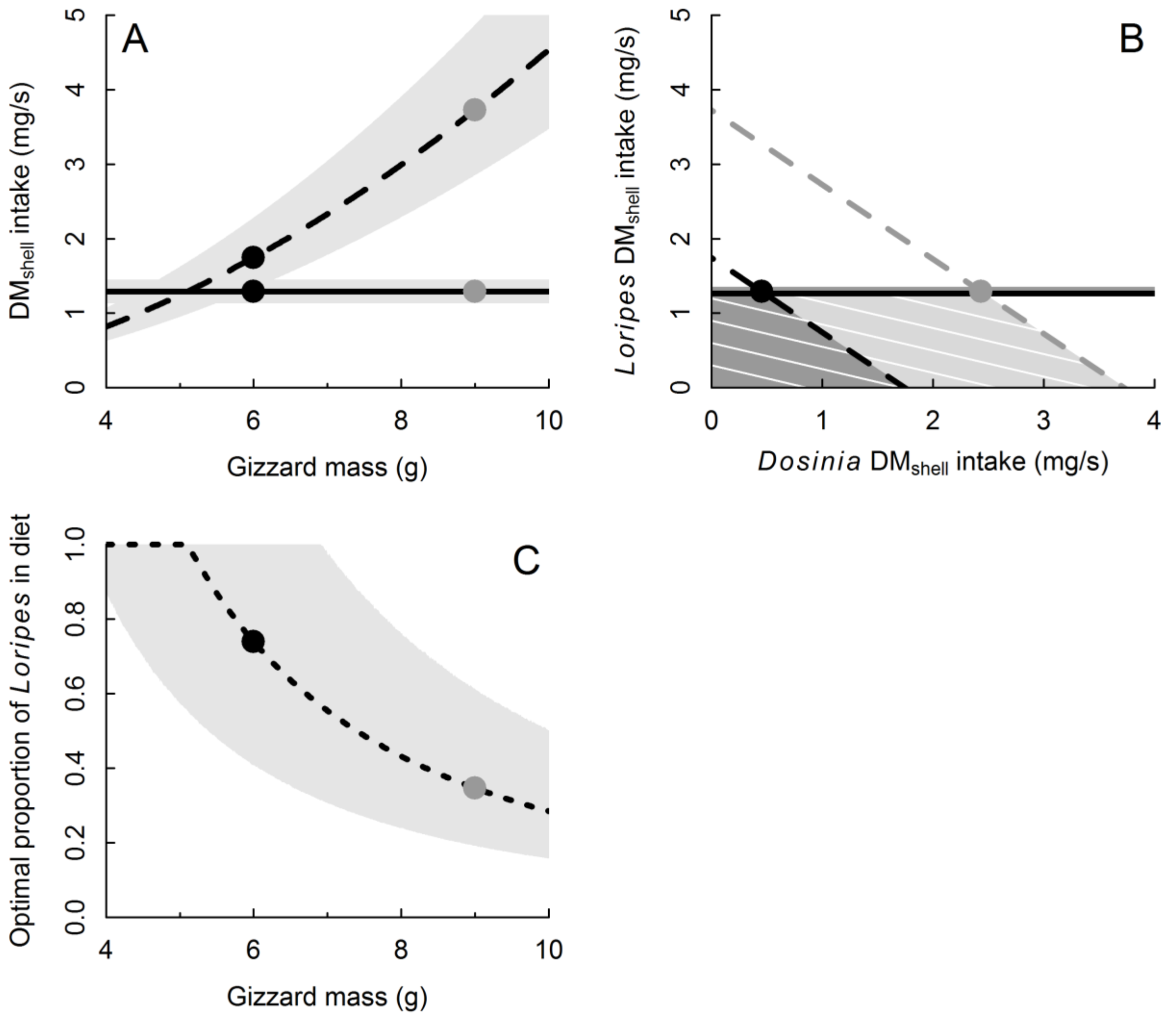

Figure S4.1. Graphical representation of the linear programming model. A) The observed relations between gizzard mass (G) and two intake constraints. Toxin constraint (q), represented by the solid line, only limits the intake of *Loripes* and is independent of gizzard mass. Digestive constraint (c), shown by the dashed line, limits the intake of both *Loripes* and *Dosinia* and increases exponentially with gizzard mass. Black dots show q and c at $G = 6$ g (comparable to small gizzard group), and grey dots show q and c at $G = 9$ g (comparable to the large gizzard group). Grey areas are estimated values ± SD. SDs were calculated as the square root of the sum of the fixed and random effect variances from the linear mixed-effect models (model 2.1 in Table 1 for *Dosinia*, intercept model on *Loripes* data for *Loripes*). B) Optimal diet choice when both *Dosinia* and *Loripes* are available *ad libitum* for a gizzard

mass of 6 g (black dot and lines) and 9 g (grey dot and lines). Solid lines show q and dashed lines show c at levels corresponding to the dots in panel A. Dark grey area shows all possible combinations under both constraints for a 6 g gizzard, light grey area for a 9 g gizzard. White lines connect points of equal energy intake rate, calculated from e_d and e_l, with increasing energy intake to the right and up. The maximum energy intake is reached where constraint lines intersect (dots). Thus, when G changes from 6 to 9 g, the digestive constraint increases (from black to grey dashed line), whereas the toxin constraint remains unchanged (black and grey solid line), leading to an increased optimal intake on *Dosinia* but not on *Loripes*. C) Expected relation between gizzard mass and the optimal proportion of *Loripes* in the diet. Dotted line connects mean predicted proportions as calculated. Grey area encloses the 95% prediction interval. Black dot shows the expected proportion at $G = 6$ g, grey dot shows expected proportion at $G = 9$ g, corresponding to the predictions in panel B.

Table S4.1. Variables and parameters used in the diet selection model.

Symbol	Value	Unit	Description
r_d	variable	mg/s	DM_{shell} Intake rate on *Dosinia*
r_l	variable	mg/s	DM_{shell} Intake rate on *Loripes*
R^*	variable		Optimal combination of r_d and r_l
G	variable	g	Gizzard mass
c	variable	mg/s	Digestive constraint, i.e. the max. DM_{shell}[a] intake rate on non-toxic prey
q	1.29	mg/s	Toxic constraint, i.e. the max. DM_{shell} intake rate on *Loripes*
e_d	0.057	mg/mg	$AFDM_{flesh}$[b] per DM_{shell} in *Dosinia*
e_l	0.163	mg/mg	$AFDM_{flesh}$ per DM_{shell} in *Loripes*

[a] Dry shell mass

[b] Ash-free dry flesh mass

References

1. Oudman T, Onrust J, de Fouw J, Spaans B, Piersma T, van Gils JA. Digestive capacity and toxicity cause mixed diets in red knots that maximize intake rate. Am Nat. 2014;183(5):650-9.
