# Supplementary material for: The Effect of Digestive Capacity on the Intake Rate of Toxic and Non-Toxic Prey in an Ecological Context
Source: PLoS One. 2015 Aug 19;10(8):e0136144. doi: 10.1371/journal.pone.0136144 (PMC4543589; doi:10.1371/journal.pone.0136144)
Supplement: S1 Table — (PDF) [file pone.0136144.s006.pdf]

S1 Table. Parameter estimates in fixed part of minimum adequate statistical models.

Parameter	Estimate	SE	DF [a]	t-value	p-value
Model 1.1: Gizzard mass ~ diet × group + (1|Bird)					
Intercept	6.09	0.38	49	16.03	<0.0001
Hard-shelled diet	1.62	0.39	49	4.16	0.0001
Group 2	0.06	0.52	4	0.11	0.91
Hard-shelled diet : group 2	1.23	0.53	49	2.32	0.02
Model 2.1: DM_{shell} [b] intake rate ~ gizzard × species + (1|Bird)					
Intercept	1.25	0.10	51	12.66	<0.0001
Large gizzard	0.07	0.10	51	0.65	0.52
Dosinia	0.75	0.23	51	3.21	0.002
Large gizzard : Dosinia	1.06	0.32	51	3.35	0.002
Model 3.1: log(DM_{shell} intake rate) ~ log(gizzard) + (1|Bird)					
Intercept	-1.21	0.17	53	-6.88	<0.0001
Log(gizzard)	1.87	0.24	53	7.83	<0.0001

NB: All models are linear mixed-effects models (function “lme” in package “nlme” in R), with bird-ID as a random effect. Parameters were estimated by maximizing the log-likelihood. In model 1.1, gizzard mass is measured in g, diet refers to either a soft or a hard-shelled diet, and group refers to experimental group (either 1 or 2, differing only in the order of the diet treatments). In model 2.1, DM_{shell} intake rate refers to dry shell-mass intake rate (mg/s), gizzard refers to the experimental treatment (being either small on a soft diet or large on a hard-shelled diet), and species refers to the prey species, being either *Loripes* or *Dosinia*. In model 3.1, log(gizzard) refers to the natural logarithm of gizzard mass (measured in g). A variance structure was incorporated in model 2.1 to correct for different variances in the *Loripes* and *Dosinia* trials.

[a] Degrees of freedom

[b] Dry shell mass
